# In reply: Additional Insights into the REPLICA-PH study

**DOI:** 10.21470/1678-9741-2023-0290

**Published:** 2024-04-15

**Authors:** Ashish Walian, Rohan Magoon, Iti Shri, Ramesh Chand Kashav

**Affiliations:** 1 Department of Cardiac Anaesthesia, Atal Bihari Vajpayee Institute of Medical Sciences (ABVIMS) and Dr. Ram Manohar Lohia Hospital, Baba Kharak Singh Marg, New Delhi, India. E-mail: drkashav@yahoo.co.in

To The Editor,

We thank Dr Karamsi for an interested reading of our article titled “Retrospective Evaluation of Platelet-Leukocyte Indices and Cardiac Surgical Outcomes in Acyanotic Heart Disease Patients with Pulmonary Hypertension (REPLICA-PH)” and would wish to respond to his observations regarding our research endeavor^[1,2]^.

Firstly, Dr Karmasi suggests an incremental value of including the hematological inflammatory indices with monocytic corpuscular lineage in the index retrospective analysis^[1,2]^. In this context, he discusses the aggregate index of systemic inflammation, AISI = “monocyte” × systemic immune-inflammation index (SII), and the systemic inflammation response index, SIRI = “monocyte” × neutrophil-to-lymphocyte ratio (NLR), where SII=NLR × platelets^[1]^. Given an ever-evolving nature of the hematological prognostication, we realize the merit of the observation, only to however elucidate that our follow-up research work in the corresponding subject features AISI and SII as independent predictors of post-cardiotomy vasoplegia syndrome in an adult cardiac surgical setting (OR; 95% CI: 1.11; 1.05-1.17, *P*<0.001 and, 1.09; 1.02-1.18, *P*=0.001, respectively)^[1,3,4]^. Indeed, the significant correlation between the mean vasoactive-inotropic score and the hematological ratio-indices under evaluation emerged as an important additional finding of the REPLICA-PH study^[2]^. We concur that employing composite indices may serve as improved representatives of perioperative inflammation, parsimonious profiling of which is at the heart of such prognostic or predictive research efforts^[1-4]^.

To Dr Karamsi’s second observation regarding our refraining to *“err on either side of the pulmonary capillary”* having referred to our study subset to be ailing from pulmonary hypertension, the lack of routine availability of data on pulmonary vascular resistance remains to be highlighted, only having had included as many as 1,040 acyanotic congenital heart disease (CHD) patients^[1,2]^. Indeed, a circumspect choice of terminology is aimed at avoiding any potential misinterpretation on the part of the readership.

Finally, we wish to humbly register our counterpoint in reference to Dr Karamsi’s concerns about excluding the multisystem syndromic cohort from our analysis^[1]^. Adequately supporting our choice of a homogeneous non-syndromic surgical subset, Zakharchenko et al.’s^[5]^ prospective observational study quite recently outlines considerable alterations in the perioperative pro-inflammatory and anti-inflammatory immune responses mounted by CHD infants with Down syndrome as opposed to those with a normal chromosomal complement^[2]^.
